# First study on the effect of transforming growth factor beta 1 and insulin-like growth factor 1 on the chondrogenesis of elephant articular chondrocytes in a scaffold-based 3D culture model

**DOI:** 10.14202/vetworld.2022.1869-1879

**Published:** 2022-07-30

**Authors:** Siriwan Tangyuenyong, Patiwat Kongdang, Nutnicha Sirikaew, Siriwan Ongchai

**Affiliations:** 1 Equine Clinic, Department of Companion Animal and Wildlife Clinic, Faculty of Veterinary Medicine, Chiang Mai University, Chiang Mai, Thailand; 2 Center of Multidisciplinary Technology for Advanced Medicine, Faculty of Medicine, Chiang Mai University, Chiang Mai, Thailand; 3 Musculoskeletal Science and Translational Research Center, Faculty of Medicine, Chiang Mai University, Chiang Mai, Thailand; 4 Thailand Excellence Center for Tissue Engineering and Stem Cells, Department of Biochemistry, Faculty of Medicine, Chiang Mai University, Chiang Mai, Thailand; 5 Center for Research and Development of Natural Products for Health, Chiang Mai University, Chiang Mai, Thailand

**Keywords:** articular chondrocyte, chondrogenesis, elephant, insulin-like growth factor 1, scaffold, transforming growth factor beta 1

## Abstract

**Background and Aim:**

Osteoarthritis (OA) is recognized as a degenerative joint disease that leads to chronic pain and low quality of life in animals. Captive elephants, the largest land mammals with a long lifespan, are more prone to develop OA due to restricted spaces and insufficient physical activity. This study aimed to investigate the effect of transforming growth factor-β1 (TGF-β1) and insulin-like growth factor 1 (IGF-1) on elephant chondrogenesis in a scaffold culture of articular chondrocytes.

**Materials and Methods:**

Elephant chondrocytes-seeded gelatin scaffolds were cultured in chondrogenic media with or without 10 ng/mL of TGF-β1 or IGF-1 alone or 5–10 ng/mL of their combination for up to 21 days. The mRNA expression of cartilage-specific anabolic genes, *ACAN* and *COL2A1*, was analyzed using a real-time reverse transcription-polymerase chain reaction. The amounts of sulfated glycosaminoglycans (sGAGs) in conditioned media and contents in cultured scaffolds were determined through dimethylmethylene blue assay. Cell morphology, accumulation of proteoglycans, and details of the cultured scaffolds were determined using hematoxylin-eosin staining, safranin O staining, and scanning electron microscopy (SEM), respectively.

**Results:**

TGF-β1 alone significantly upregulated *ACAN* gene expression but not *COL2A1*, while IGF-1 alone did not enhance both *ACAN* and *COL2A1* genes. The combination significantly upregulated both mRNA expression levels of *ACAN* and *COL2A1* gene at day 14. The sGAGs accumulation and contents in the treatment groups, except IGF-1 tended to be higher than the controls, concomitantly with the production of the extracellular matrix, showed the formation of a cartilage-like tissue through histological and SEM analyses.

**Conclusion:**

Together, our results suggest that the single treatment of TGF-β1 has a selective effect on *ACAN* gene, while the combined growth factors seem to be an advantage on elephant chondrogenesis. This three-dimensional culture model is probably helpful for developing cartilage regeneration *in vitro* and is further applied in tissue engineering for OA treatment *in vivo*.

## Introduction

Osteoarthritis (OA), apparently characterized by cartilage destruction, is a common joint disease caused by morbidity or mortality in captive elephants [1]. Articular cartilage is composed of a dense extracellular matrix (ECM) with a small percentage of highly specialized cells called chondrocytes. Chondrocytes are cartilage cells that play a crucial role in regulating cartilage matrix synthesis and maintenance of tissue homeostasis. The ECM of articular cartilage synthesized by chondrocytes, composes of hyaluronan, predominant type II collagen, and aggrecan, which major proteoglycan consists of various sulfated glycosaminoglycans (sGAGs). Unfortunately, the articular cartilage limits self-repair [2] due to lack of vascular, neural, and lymphatic networks; thus, repeated damage results in irreversible cartilage degeneration. The principles of OA treatment include alleviation of pain and improvement of joint function in elephants, similar to humans. To date, the current approach for regenerative treatment in OA humans is cartilage tissue engineering. However, the effective therapeutic regimen for OA elephants is still limited and cartilage tissue engineering does not appear to be researched or used in elephants.

Tissue engineering has gained increasing interest and developed to apply for cartilage restoration in humans and animals [3, 4]. Several studies have reported appropriate cell sources, biocompatible and biodegradable scaffolds, effective growth factors, and suitable techniques to generate cartilage tissue *ex vivo* for *in vivo* implantation [5–7]. The natural polymer, such as a gelatin scaffold, provides a suitable three-dimensional (3D) microenvironment to contribute full-chondrogenic differentiation to chondrocyte phenotype, resulting in enhanced ECM production [8]. By contrast, the passaged chondrocytes by expansion in two-dimensional (2D) culture induce dedifferentiation leading to decreased cartilage matrix synthesis [9]. Growth factors are the critical factors that assist cell differentiation to a specific phenotype. In cartilage metabolism, the anabolic and catabolic processes of chondrocytes are proceeded by some growth factors. The transforming growth factor-b (TGF-β) superfamily consisting of more than 30 members is associated with cell growth, proliferation, differentiation, migration, and apoptosis, including cartilage matrix synthesis and degradation [10]. There are three isoforms of TGF-β in mammals, TGF-β1, TGF-β2, and TGF-β3. The TGF-β1, one of the most studied in the musculoskeletal system, plays a key role in chondrogenesis [11]. In 3D system by pellet and scaffold cultures, TGF-β1 enhances chondrogenic differentiation and proliferation and increases collagen and proteoglycan synthesis of ECM in humans, bovines, and rabbits [12–14]. Furthermore, insulin-like growth factors (IGFs) in two distinct forms, IGF-1 and IGF-2, play a crucial role in tissue metabolism [15]. The IGF-1 promotes cell proliferation and differentiation of chondrocytes and stimulates cartilage matrix production in a 3D culture of various species [5, 6, 16]. In addition, it has been well reported that using 2D or 3D cultures, TGF-β1 and IGF-1 alone or in combination can enhance the chondrogenic differentiation and cartilage matrix synthesis of chondrocytes from human and bovine [17, 18]. In elephants, there are few studies with regard to cartilage metabolism. A previous study conducted by Sirikaew *et al*. [1] in elephant chondrocytes revealed that pro-inflammatory mediators, such as interleukin (IL)-1b, IL-17A, and tumor necrosis factor-alpha, could activate the expression of matrix metalloproteinases, resulting in cartilage degradation, which was suppressed by common pharmacological or herbal medications used to treat OA in humans**.** Till now, a report regarding the effect of TGF-β1 and IGF-1 on chondrogenesis in elephant articular chondrocytes (ELACs) is still rare.

This study aimed to investigate chondrogenesis in ELACs underlying the stimulation of TGF-β1 and IGF-1 on cartilage-specific anabolic gene expression and ECM biomolecule synthesis using scaffold-based 3D culture. In addition, we evaluate whether the 3D culture systems provide an appropriate model for the study of TGF-β1 and IGF-1 and their effects on chondrocyte culture in an Asian elephant. The results gained from this study will provide a more fundamental understanding of articular cartilage metabolism and support further research, including the development of regenerative treatment, leading to improved quality of life, and the long lifespan of elephants.

## Materials and Methods

### Ethical approval

All procedures and animals used in this study were approved by the Institutional Animal Care and Use Committee, Faculty of Veterinary Medicine, Chiang Mai University, Chiang Mai, Thailand (FVM–ACUC; Ref. No. R17/2559).

### Study period and location

The study was conducted from February 2019 to October 2021. Experiments were done at Thailand Excellence Center for Tissue Engineering and Stem Cells, Department of Biochemistry, Faculty of Medicine, Chiang Mai University, Chiang Mai, Thailand.

### Preparation of primary ELACs

Elephant articular cartilages from a stillborn elephant calf, which was dead by dystocia in an elephant camp, Chiang Mai, Thailand, were collected aseptically from the weight-bearing areas of the clinically normal femorotibial joint without pathological lesions within 6–8 h postmortem under owner’s authorization. ELACs were isolated by overnight digestion with 2 mg/mL type II collagenase (Worthington, Biochemical Corp., Freehold, NJ, USA) at 37°C. The passage 0 chondrocytes were investigated their morphology using an inverted phase-contrast microscope (Motic AE2000, Motic Microscopes, Schertz, TX, USA) and obtained photographs with Canon EOS 650D camera. After washing with phosphate-buffered saline (PBS), the ELACs were grown as monolayers in Dulbecco’s Modified Eagle’s Medium (DMEM; Gibco, Waltham, MA, USA) containing 10% v/v fetal bovine serum (FBS; Gibco), penicillin (100 U/mL; Gibco), and streptomycin (100 mg/mL; Gibco) in a humidified incubator at 37°C with 5% CO_2_ until 80% confluence. The primary ELACs were grown and expanded up to passage 4 (P4) for use in the scaffold-based 3D culture of this study. Each passage was cultured and trypsinized at 80% confluence.

All chemicals used in this study were purchased from Sigma Chemical Company (Sigma-Aldrich, St. Louis, MO, USA) unless otherwise stated.

### Scaffold culture and growth factor treatment of ELACs

The gelatin-based scaffold (Spongostan Standard; Johnson and Johnson, Germany) was cut into 5 mm × 2.5 mm circles using a metal punch. All scaffolds were sterilized at 121°C, 15 psi pressure, and 25 min using an autoclave and then were dried at 60°C for 2–3 days using a hot air oven. Before chondrocyte seeding, the scaffolds were transferred to a sterile Petri dish and presoaked with 10% FBS-DMEM. After transferring scaffolds to a blank 24-well plate, elephant chondrocytes (1 × 10^6^ cells suspension in 20 μL culture media) were directly seeded onto the upper surface of the scaffolds. Then, they were incubated at 37°C with 5% CO_2_ for 4 h to let chondrocytes diffuse and attach to the scaffolds. Next, 1 mL of culture medium/well was added, and overnight incubation was conducted. Finally, the cellular scaffolds were transferred to a new 24-well plate to begin a 21-day culture. Each scaffold was treated and incubated with 1 mL of chondrogenic media, DMEM supplemented with 10% FBS, 1× insulin–transferrin–selenium (10 mg/mL insulin, 5.5 mg/mL transferrin, and 5 ng/mL selenium; PAA Laboratories, Austria), 25 mg/mL ascorbic acid, and 10^−7^ M dexamethasone [19], containing chondrogenic growth factors, 10 ng/mL recombinant human TGF-β1 (PeproTech Asia, Israel), recombinant human IGF-1 (PeproTech Asia), or 5–10 ng/mL of their combination or without any growth factor as control, at 37°C in a humidified atmosphere of 5% CO_2_ for up to 21 days. Chondrogenic media were changed and collected every 2–3 days to evaluate the cytotoxic effect and sGAGs release using lactate dehydrogenase (LDH) and dimethylmethylene blue (DMMB) spectrophotometric assays, respectively. The scaffolds were harvested once a week for anabolic gene expression analysis using quantitative real-time reverse transcription-polymerase chain reaction (RT-PCR) and at day 21 for sGAGs content, histological analysis, and scanning electron microscopy (SEM) examination.

### Evaluation of the cytotoxic effect

The cytotoxic effect of treatment conditions on the elephant chondrocytes-seeded 3D scaffold was determined by the level of cytoplasmic LDH enzyme released into the scaffold culture media. The procedure was performed as previously described by Megraw [20].

### Evaluation of sGAGs

The amounts of sGAGs in conditioned media and the contents of sGAGs in the cultured scaffold were measured using DMMB assay as previously described by Farndale *et al*. [21]. Briefly, a cation in the 1,9–dimethylmethylene dye bound to sGAGs induces metachromasia enabling rapid detection of sGAGs in solution. Chondroitin 6-sulfate (CS-C: 0–30 mg/mL) as a known concentration standard, and unknown sGAGs amounts (50 mL) of conditioned media or papain digest solution of the scaffold were placed in 96-well plate, and 200 mL of DMMB was added. The solution was mixed using a shaker, at 30× *g*, for 1 min. The absorbance values were then measured by a microplate reader spectrophotometer (Multiskan, Thermo Scientific, USA) at 540 nm. The levels of sGAGs in conditioned media were calculated using a relative standard curve.

### Analysis of chondrogenic anabolic gene expression

The expression of the chondrogenic anabolic genes in scaffolded 3D cultures was investigated on days 7, 14, and 21 using real-time RT-PCR in accordance with the manufacturer’s protocol. Then, cultured scaffolds were suspended in a lysis solution for RNA extraction. The total RNA of the cultured scaffolds using the illustra RNAspin Mini RNA Isolation Kit (GE Healthcare Life Sciences, UK) was converted to cDNA using the ReverTra Ace qPCR RT Master Mix (Toyobo, Japan). To determine the chondrogenic gene expression, specific primers and SensiFast SYBR No-ROX reagent (Bioline, UK) were used for real-time PCR on 7500 Fast Real-time PCR system (Applied Biosystems, Foster City, CA, USA). The African elephant primer sequences were designed from the association with GenBank accession numbers using the NCBI Primer-BLAST tool [22] as follows: aggrecan core protein (*ACAN*; XM_023553039.1): forward 5′-ACTTCCGCTGGTCAGATGGA-3′; reverse 5′-TCTCGTGCCAGATCATCACC-3′: collagen type II (*COL2A1*; XM_003405744.2): forward 5′-CTCGTGGCAGAGATGGAGAG-3′; reverse 5′-CACCAGGCTCACCAGGATTG-3′: glyceraldehyde-3-phosphate dehydrogenase (*GAPDH*; XM_010590823.2): forward 5′-CCAGCTAGGGCTCCTTTCTTT-3′; reverse 5′-CCGCAAATGAAACCTTCCCG-3′. The mRNA values of samples were calculated using the 2^−∆∆Ct^ method relatively to untreated control and normalized to the expression of the *GAPDH* gene as a housekeeping gene as previously described by Boykiw *et al*. [23].

### Histological examination

On day 21, the ELACs cultured on the scaffolds were washed with PBS and fixed in 4% paraformaldehyde (RCI Labscan, Thailand). All scaffold samples were sent to the Department of Pathology, Faculty of Medicine, Chiang Mai University, Thailand, for paraffin embedding, tissue blocking, microtome sectioning, and hematoxylin-eosin (H&E) staining. To determine the remaining sGAGs in cultured scaffolds, the unstained sections were then used in safranin O staining. Next, the sections were deparaffinized, rehydrated, and stained with 0.1% safranin O staining solution for 1 h at 26°C. After dehydration, slide mounting and drying were conducted.

### Scanning electron microscopic examination

On day 21, the cultured scaffolds were washed with PBS and fixed in 3% glutaraldehyde in 0.1M PBS overnight at 4°C. After washing with a mixture of 0.1M PBS and 2% osmium at 26°C for 1 h, they were dehydrated through a series of increasing concentrations of ethanol (50%, 75%, 90%, and 100%) until they reached their critical point of drying using CPD 7501. Dry scaffolds were sputter-coated with SPI-Module Sputter Coater (SPI Supplies, PA, USA) before observation under a scanning electron microscope JSM 661 LV apparatus (Jeol, Japan) at 15 kV [24].

### Statistical analysis

All results were expressed as means ± standard error of the mean from three or four independent experiments. The differences in means of sGAGs accumulation, *ACAN* and *COL2A1* gene expression between control and treatment groups at each time point were analyzed using a two-way analysis of variance (ANOVA). In addition, if the outcomes, including sGAGs content and LDH activity measured at the end of the experiment, the analysis was performed using one-way ANOVA. Multiple comparisons were undertaken using Benjamini–Hochberg’s test. The level of significance was set as α = 0.05. All analyses were conducted using R software, v.4.1.1 (R Foundation for Statistical Computing, Austria).

## Results

### Morphology of elephant chondrocytes in monolayer and 3D cell cultures

**Figure-1:**
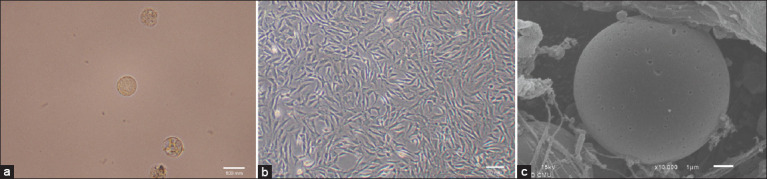
Morphology of the elephant articular chondrocytes (ELACs) in monolayer and scaffold-based cultures by microscopic examination. In monolayer culture, (a) freshly isolated ELACs at 400× magnification; (b) the third passage ELACs at 100× magnification; (c) In 3D, ELACs embedded in the scaffold at 21 days, 10,000× magnification. Scale bar = 1 mm.

Microscopic pictures of the elephant chondrocytes in monolayer and scaffold-based 3D cultures are shown in Figure-1a-c. In the monolayer culture of the control group, the elephant chondrocytes, freshly isolated from articular cartilage tissue, exhibited a characteristic of spheroid morphology under an inverted phase-contrast microscope (Figure-1a). After two to four culture passages, the chondrocytes transformed their appearances from a round shape to fibroblast-like cells as a spindle (Figure-1b). After the monolayer elephant chondrocytes were grown in scaffold-based 3D culture for 21 days, a scanning electron micrograph of the elephant chondrocyte returned to the spherical-shaped cell, embedded in ECM on the gelatin scaffold (Figure-1c).

### Chondrogenic anabolic gene expression and ECM synthesis in 3D culture

**Figure-2:**
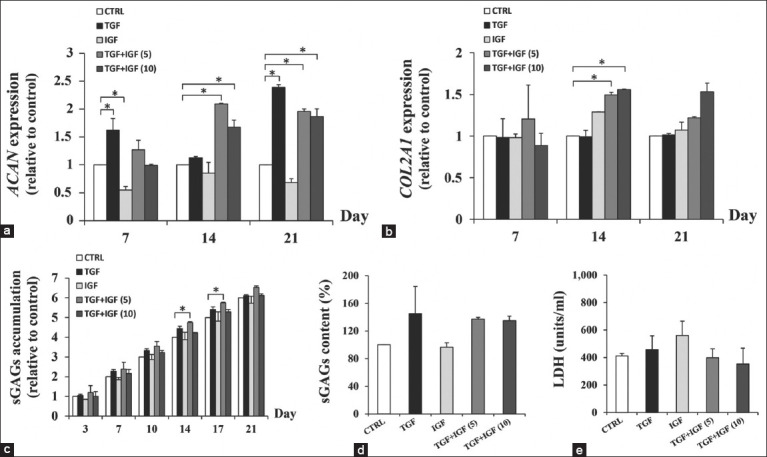
Growth factors enhanced *ACAN* and *COL2A1* expression and sulfated glycosaminoglycans (sGAGs) synthesis in scaffold-based cultures of elephant articular chondrocytes (ELACs). The ELACs embedded in the scaffold were cultured for 21 days in the absence (CTRL) or presence of either transforming growth factor-β1 (TGF-β1) or insulin-like growth factor 1 (IGF-1) (each at 10 ng/mL) alone and combined; each at 5 ng/mL = TGF + IGF (5), and each at 10 ng/mL = TGF+IGF (10). The expression of (a) *ACAN*; (b) *COL2A1* genes; (c) The cumulative amounts of sGAGs in conditioned media; (d) The contents of sGAGs in the cultured scaffold; (e) Cytotoxicity by lactate dehydrogenase activity. Asterisks denote significant differences between control and treatment groups (p < 0.05).

Chondrogenic anabolic gene expressions, including *ACAN* and *COL2A1* genes, were determined weekly for 21 days of the culture (Figure-2a and b). The expression of the *ACAN* gene was significantly upregulated in the elephant chondrocytes-seeded scaffolds treated with TGF-β1 alone compared with those of the controls (p = 0.0017 and p < 0.0001 on days 7 and 21, respectively) while low *ACAN* expression was found in IGF-1 treated group (Figure-2a). The level of *COL2A1* gene expression did not show a significant response to the single treatment of these growth factors (Figure-2b). In the combination of TGF-β1 and IGF-1, 5 and 10 ng/mL of combined treatment showed significant higher level of *ACAN* expression on days 14 (p < 0.0001 and p = 0.0007) and 21 (p < 0.0001 and p < 0.0001), whereas the *COL2A1* gene expression was significantly increased on day 14 only (p = 0.0487 and p = 0.0487) when compared with those of the controls. In comparison between single and combined treatments at days 14, 5, and 10 ng/mL of combined treatment had the levels of *ACAN* expression higher than those of TGF-β1 (p < 0.0001 and p = 0.0032) and IGF-1 treated alone (p < 0.0001 and p = 0.0001). For *COL2A1* gene, 5 and 10 ng/mL of combined treatment showed the expression higher than those of TGF-β1 (p = 0.0487 and p = 0.0487). To compare combined treatments on day 14, only *ACAN* gene expression in 5 ng/mL of the combination was significantly higher than those of 10 ng/mL.

The production and release of sGAGs into the media were determined by the levels of sGAGs accumulation in the cultured media and sGAGs content in the scaffolds for 21 days of the culture (Figure-2c and d). The accumulation levels of sGAGs gradually increased for up to 21 days of the culture in all groups. On days 14–17, only the combination of 5 ng/mL TGF-β1 and IGF-1 treated scaffolds had higher cumulative amounts of sGAGs than those in controls significantly (p = 0.0448 and p = 0.0408). Nevertheless, there was a slight and non-significant increase in sGAGs level was found in 5 ng/mL combination group than in control on day 21. In 10 ng/mL combined growth factor, the trend of sGAGs accumulation was higher than that of the control group (Figure-2c). The sGAGs contents in treatment with alone TGF-β1 and growth factor combination slightly increased compared with those of the control (Figure-2d). The cytotoxic effect of the treatment conditions on the elephant chondrocytes-seeded 3D scaffold was determined in the conditioned media on day 21 (Figure-2e). Neither the scaffold cultures treated with TGF-β1 or IGF-1 alone nor the combination at the concentration up to 10 ng/mL showed significant changes in the LDH activity compared with the control.

### ECM synthesis: Microscopic examinations of the scaffolds in 3D cultures

**Figure-3:**
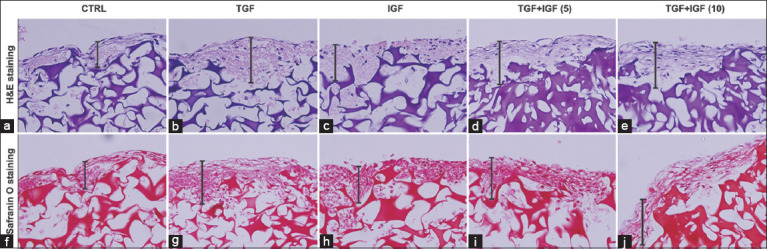
Growth factors enhanced extracellular matrix synthesis (ECM) synthesis in histological staining of scaffold-based cultures of elephant articular chondrocytes (ELACs). (a-e) H&E and (f-j) safranin O staining of the scaffolds embedded with ELACs. The vertical bars represent the areas of ECM formation. The histological image in each group is a representative of three independent slides. Full images with scale of histological staining are provided in Supplementary Information.

**Figure-4:**
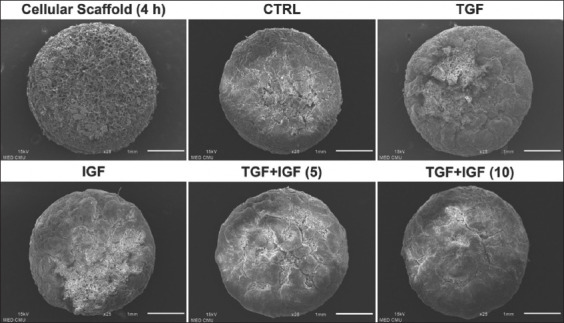
Growth factors enhanced extracellular matrix synthesis in scanning electron micrographs of scaffold-based cultures of elephant articular chondrocytes. 25× magnification, scale bar = 1 mm.

**Figure-5:**
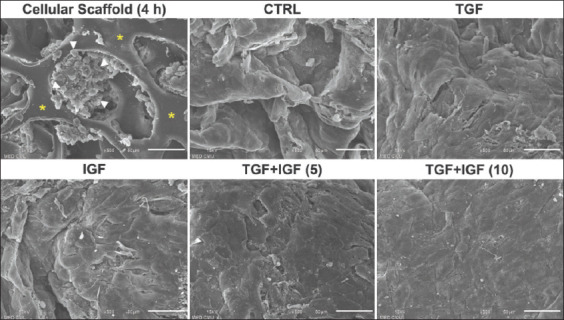
Growth factors enhanced extracellular matrix synthesis in scanning electron micrographs of scaffold-based cultures of elephant articular chondrocytes (ELACs). 500× magnification, scale bar = 50 μm. Asterisks denote the scaffold structure. Arrowheads indicate the ELACs.

Histological characteristics regarding the growth of chondrocytes and synthesis of ECM in the 3D cultured scaffolds with or without treatments of growth factors were investigated under microscopic examinations (Figures-3–5). The chondrocyte morphology and proteoglycan synthesis in the scaffolds assessed by H&E and safranin O staining, respectively, are shown in Figure-3. The H&E staining and flattened chondrocytes were observed along the surface of the scaffolds. In the growth factor-treated scaffolds, the formation of a cartilage-like ECM containing chondrocytes at the scaffold surface was increased compared with the control (Figure-3 and Panels a-e). The staining of safranin O revealed the increment of sGAGs formation in the scaffolds treated with growth factors in comparison with the untreated control (Figure-3 and Panels f-j).

To assess cell morphology and ECM synthesis, scanning electron micrographs of the chondrocytes-embedded scaffolds from the control and growth factor-treated groups are shown in Figures-4 and 5. The SEM image of the untreated cellular scaffold, which was shortly present at 4 h after cell seeding, displayed a coarse-textured structure with many internal pores containing variable-shaped chondrocytes at the scaffold surface (Figures-4 and 5). On day 21 of culture, the microscopic consistency of the cellular scaffolds treated with growth factors seemed more compact when compared with both the 4-h- and 21-day-cultured cellular scaffolds from the untreated control groups (Figure-4). High-magnification assessment of the ECM in the chondrocytes-embedded scaffolds for 21 days of culture showed that the 4-h-cultured cellular scaffold of the control group was highly porous. However, the cellular scaffolds treated with growth factors were denser with a continuous layer of hyaline cartilage-like tissue throughout the surface of the scaffolds. The scaffold surfaces of the combination treatments seemed smoother than those of single-treated scaffolds (Figure-5).

## Discussion

OA, one of the most common joint diseases that influence elephants’ life quality, is markedly characterized by irreversible articular cartilage deterioration [25]. Articular cartilage has a limitation in the ability of self-regeneration [2, 8]. At present, tissue engineering has been an alternative treatment in OA humans by producing hyaline cartilage composed of combining cells, scaffolds, and growth factors *ex vivo* for *in vivo* cartilage restoration. TGF-β1 has been known as a potent stimulator in the proliferation and differentiation of chondrocytes and induces the synthesis of cartilage matrix, including aggrecan, proteoglycan, and type II collagen in humans and animals [26, 27]. IGF has been reported to enhance chondrocyte proliferation, promote differentiation, and increase ECM production *in vitro* and *in vivo* models [6, 28]. However, knowledge pertaining to chondrogenesis in elephants is still limited *in vitro* and *in vivo*.

This study focused on the influence of TGF-β1 and IGF-1 on elephant chondrogenesis. The anabolic genes, *ACAN* and *COL2A1*, defined as chondrogenic markers, encode aggrecan core protein, and type II collagen which are predominantly composed in ECM, respectively [29]. Thus, these genes have an essential role to play in the regulation of chondrogenesis. The increase in their expression levels appears during cartilage formation. A previous study demonstrated that TGF-β1 treatment in human adipose-derive mesenchymal cells (MSCs) increased mRNA and protein expression of aggrecan and collagen type II in monolayer culture [28]. In 3D pellet cultures, high levels of aggrecan and pro-collagen type II protein expression in human chondrocytes [18] and upregulated mRNA expression of *ACAN* and *COL2A1* were found in mice MSCs treated with TGF-β1 [16]. For treatment by IGF-1, the previous studies [5, 6, 30, 31] showed that aggrecan and collagen type II protein expression were elevated in MSCs and chondrocytes of various species in pellet and scaffold cultures as well as cartilage explant model. The mRNA expression of *ACAN* and *COL2A1* was upregulated in MSCs treated with IGF-1 using monolayer and pellet cultivation [16, 28, 31]. In the present study, the expression of *ACAN* gene in the ELACs-seeded scaffolds was upregulated in TGF-β1 treatment, but not *COL2A1*. In contrast, the IGF-1 treatment in our study did not increase both gene expressions. This indicated that *ACAN* and *COL2A1* genes had selectivity in responsiveness to types of growth factors and animal species.

Along with TGF-β1 and IGF-1 treatment, this study found that the combined treated scaffolds had *ACAN* and *COL2A1* gene expression higher than those of TGF-β1 or IGF-1 treated alone in some period of time (day 14). Similarly, the previous studies reported that mRNA and protein expressions of collagen type II and aggrecan in the stimulation of both TGF-β1 and IGF-1 were higher than TGF-β1 or IGF-1 treated human chondrocytes and mice MSCs in pellet cultures [16, 18, 32]. Moreover, there was greater protein expression of collagen type II and aggrecan, and greater mRNA expression of *ACAN* and *COL2A1* following TGF-β1 and IGF-1 combined treatment, compared with either TGF-β1 or IGF-1 treated human MSCs in monolayer culture [28]. Another study revealed that combining TGF-β1 with IGF-1 maintained type II collagen mRNA expression and enhanced the overall total cartilage growth in rabbit MSCs compared with the single treatment [30]. Thus, in conjunction with those of the previous studies [16, 18, 28, 30, 32], our results indicate that the combined treatment of TGF-β1 and IGF-1 seem to be beneficial in stimulating the activity of cartilage-specific anabolic genes in elephant chondrogenesis.

According to the different gene expression profiles in responsiveness to the combined treatments, the characteristic of *COL2A1* gene expression on day 21 became different from day 14. Regarding the *ACAN* gene expression, this study noted that its responses to those two growth factors were dissimilar to that of the *COL2A1* gene. These findings may suggest that the different gene expression patterns not only depend on type of gene but also rely on the duration time of the experiment.

Signaling pathways in chondrogenesis related to growth factors has been described in previous studies [33–35]. TGF-β plays a key role in cell proliferation, differentiation, and ECM production by activating the Smad 2/Smad 3 and also signals through ERKs, JNKs, and MAPK protein p38 [33–35]. IGF-1 acts through PI3K/Akt/mTOR and MEK/ERK pathways [36]. The previous study conducted by Sarenac *et al*. [37] reported that IGF-1 suppressed TGF-β/Smad signaling of fibrosis in human keratocytes by blocking Smad 3 phosphorylation and nuclearization. In the present study, our result showed decreased *ACAN* expression in the combination of TGF-β1 and IGF-1 when compared to TGF-β1 alone. This may result from inhibition of TGF-β/Smad signaling by IGF-1. Nevertheless, treatment with IGF-1 alone appeared to reduce *ACAN* expression, suggesting that IGF-1 selectively interferes some pathways involved in the basal level of this gene expression. However, this situation does not influence the *COL2A1* gene. It is, therefore, interesting to further investigate the actual signaling pathways of IGF-1 involved in ECM synthesis in elephant chondrocytes.

 Our measurements of the production and release of sGAGs in scaffold cultures demonstrate that combined treatment might induce chondrogenic anabolic genes to synthesize cartilage matrix rather than those of either TGF-β1 or IGF-1 stimulation. This interpretation is supported by our results that showed the cumulative amounts of sGAGs in conjunction with *ACAN* and *COL2A1* gene expression were higher in the combined treated scaffolds than those of the control and single treatment. Concomitantly, the slight increased formation of hyaline cartilage-like ECM in H&E, safranin O staining, and SEM appeared in combined TGF-β1 and IGF-1 treatment. These results of the present study are in accordance with the previous findings that the level of aggrecan and collagen type II protein and gene expression was greater, as well as the staining of proteoglycans and collagen type II both *in vitro* and *in vivo* using cell-seeded scaffold transplantation, compared with controls and TGF-β1 or IGF-1 treated alone [28, 30]. However, the amounts of sGAGs contents in the combined treated scaffolds slightly increased, whereas sGAGs accumulation released into culture media tended to be higher than the control at the end of the experiment. This result suggests that sGAGs contents in the scaffolds may be newly synthesized in a small amount by chondrocytes; this is consistent with appearances of newly formed sGAGs at the surface of the scaffolds in H&E and safranin O staining. Besides, it is possible that the sensitivity of the DMMB technique used for sGAGs determination in this study may not be high enough to discriminate the difference in the content of newly synthesized sGAGs in the scaffolds among groups. Furthermore, the greater sGAGs released into culture media accompanied by the slightly increased sGAGs content in the scaffolds may imply the increase in the turnover rate of cartilage metabolism regarding high ECM degradation and production in the combined treatment groups. In addition, 21-day cultivation in our study may perhaps provide enough time for growth and ECM synthesis by chondrocytes, leading to a high baseline in the control group. Thereby, the modified DMMB assay may be applied to improve assay sensitivity [38, 39].

Regarding the cell morphology in the present study, the elephant chondrocytes freshly isolated from articular cartilage tissue showed spheroid morphology. After passage in monolayer culture, they transformed into fibroblast-like appearance leading to a reduction in chondrogenic properties. Our result proved that they returned to a spherical shape after growth in scaffold culture. These demonstrate that the mature chondrocytes from cartilage tissue dedifferentiated to fibroblast-like chondrocytes in the serial passage of 2D culture and subsequently redifferentiated to the chondrocyte phenotype in scaffold-based 3D culture with growth factor supplementation. Our interpretation is concomitant with previous studies [40, 41] that reported that fibroblast-like chondrocytes showed redifferentiation by TGF-β1 and combined TGF-β1 with IGF-1 supplemented culture in human chondrocytes. Similarly, another study revealed that the dedifferentiated cells returned to the chondrocyte phenotype through the facilitation of a 3D system [40]. Furthermore, the previous research has pointed out that the chondrocytes showed spherical morphology in 3D scaffold culture using collagen sponge [42]. In H&E staining, the combination of TGF-β1 and IGF-1 treatment in this study showed more cell growth and distribution in the edge of scaffolds than in the control. This finding was similar to previous studies; which reported that plentiful growth and cell penetration was found in human chondrocytes in TGF-β3 [8] and in TGF-β1 and IGF-1 treated gelatin scaffolds [13]. Further evidence of TGF-β1 and IGF-1 effects from several researchers unveiled that their combined treatment promoted proliferation and chondrogenic differentiation of MSCs and chondrocytes [13, 16, 28, 30]. Altogether, we postulate that TGF-β1 and IGF-1 may act in combination to induce the growth and differentiation of elephant chondrocytes during chondrogenesis.

Regarding H&E staining, the formation of a cartilage-like ECM by chondrocytes at the scaffold surface was confirmed by safranin O staining to prove that structure was really sGAGs as a major component of cartilage matrix. Remarkably, neo-synthesis of cartilage matrix only appeared around the surface of the scaffold because ECM was substantially produced by chondrocytes and tightly covered the upper surface of the scaffold. Consequently, the diffusion of oxygen, nutrients, signaling molecules, and waste products were not carried toward the scaffold’s center to nourish the chondrocytes that dwelled in the inner areas, resulting in cell death [8, 42].

Our study encountered some limitations that the safranin O staining used for sGAGs detection may not probably be highly sensitive for our study. We investigated the expression of the *COL2A1* gene as one chondrogenic marker for chondrogenesis; notwithstanding, the collagen content in the scaffold was not determined because the scaffold type used in our study is gelatin, which is a hydrolyzed product from collagen. Thus, immunohistochemistry is essential to confirm the results of gene expression and to clearly determine proteoglycan and collagen as the main component of the cartilage matrix. Unfortunately, the species-specific antibody used to identify proteoglycan and collagen type II for elephants is not available. However, we had ever investigated sGAGs and collagen type II by applying antibodies of other species (e.g., porcine, goat, and human), but it was not successful. This suggests that the antibodies for those animal species have no cross-reactivity with the protein in elephant. Therefore, improvement of efficiency in the measurement of cartilage matrix components is required for further study.

The gelatin scaffold has been widely used for cartilage regeneration and tissue engineering [43–46] and is naturally produced from the hydrolysis of collagen type I, leading to low immunogenicity and cost [47]. Moreover, its porous structure facilitates infiltration and nutrient supply of chondrocytes [48, 49]. In our study, a commercial porcine gelatin absorbable sponge applied for hemostasis was used as a gelatin scaffold to mimic a natural ECM for elephant chondrocyte culture. According to LDH activity, the scaffolds and treatment conditions in this study had no cytotoxic effect on the chondrocytes. Therefore, our 3D cultured model using the gelatin-based scaffold is suitable for studying elephant cartilage metabolism and tissue regeneration. This interpretation is supported by a plethora of previous studies [13, 50, 51] that recommended that Spongostan was an appropriated 3D matrix in chondrocyte culture. The main advantage of 3D culture is that it provides a proper microenvironment for chondrocytes [52], and the cells *in vivo* are surrounded by other cells and the ECM [8]. Moreover, the 3D culture encourages redifferentiation of cells *in vitro* and facilitates chondrocytes to maintain chondrogenic properties [8]. However, its limitation in stabilizing the chondrogenic potential of cells residing in the central part of the scaffold has been realized. Throughout the decade, various scaffolds seeded with cells, growth factors, and delivery vehicles were developed to improve the scaffolds’ affinity, loading efficiency, and diffusibility for cartilage regeneration. Recently, a study reported that the polydopamine/poly(e-caprolactone) scaffold coated with IGF-1 laden poly(lactic-co-glycolic acid) nanoparticles showed higher cell adhesion, proliferation, differentiation, and glycosaminoglycan content, including chondrogenic protein and anabolic gene expression compared with other scaffolds [30].

Both TGF-β1 and IGF-1 used in the present study were recombinant human TGF-β1 and IGF-1 proteins, respectively. Our findings demonstrate that human TGF-β1 and IGF-1 are practically applied to analyze chondrogenic anabolic gene expression and ECM synthesis in elephants. However, the study involved in TGF-β1 and IGF-1 effects on chondrogenesis has been extremely limited in elephants. Surprisingly, the structure of both elephant TGF-beta type I (TGFBR1) and TGFBR2 receptors is 90% homologous to human proteins [53]. For IGF-1 receptor (IGF1R), the protein sequences of elephant IGF1R are 90% similar to humans [53]. Therefore, the homology of the receptors between these two species suggests that the elephant TGF-β and IGF-1 receptors could recognize human TGF-β1 and IGF-1, respectively. Unfortunately, the structural similarities of those receptors are identified between African elephants (*Loxodonta africana*) and humans (*Homo sapiens*), but there are no data available on Asian elephants (*Elephas maximus*). Nevertheless, 90–100% similarity of TGF-β1 protein is reported between Asian and African elephants [53]. Moreover, the limitation on the acquisition of species-specific growth factors may influence on stimulating efficacy of TGF-β1 and IGF-1 in elephant chondrogenesis. Lack of knowledge in elephant cartilage metabolism concerning the suitable type, effective dosage, and species-specificity of growth factor; therefore, it needs to be studied further.

The current research innovation is the use of elephant chondrocytes and scaffolds culture model to study the effect of TGF-β1 and IGF-1 on chondrogenesis by focusing on chondrogenic activity associated with cartilage matrix production. The present study results suggest that the elephant chondrocytes in 2D toward 3D cultured system demonstrated alteration of cellular properties to chondrogenic phenotype eventually. Furthermore, the specific genes related to chondrogenic differentiation, such as *SOX9* and *RUNX2* genes, are suggested to further determine the genotypic influence of these growth factors.

## Conclusion

This study demonstrated for the first time that the TGF-β1 and IGF-1 in single and combined treatment have a selective effect on chondrogenic-associated genes at the specific time point in chondrogenic differentiation and ECM synthesis in Asian elephants. In addition, our scaffold-based 3D system is appropriate to use as a fundamental model that could be useful to develop and apply for further study in elephant cartilage regeneration *in vitro* and *in vivo* for OA in elephants.

## Authors’ Contributions

ST and SO: Conceptualization, project administration., ST and NS: Data curation. ST: Formal analysis. SO: Investigation, Supervision. ST, PK, and NS: Methodology. ST and PK: Software and Visualization. ST, PK, and SO: Validation and Writing - review and editing. ST: Writing - original draft. All authors have read and approved the final manuscript.
